# Sleep Disorders in South–South Latino Migrants: The Role of Acculturation in the Subjective Assessment of Insomnia Symptoms

**DOI:** 10.3390/healthcare13080904

**Published:** 2025-04-15

**Authors:** Alfonso Urzúa, Javier Torres-Vallejos, Diego Aragón-Caqueo

**Affiliations:** 1 Escuela de Psicología, Universidad Católica del Norte, Antofagasta 1270709, Chile; 2 Escuela de Psicología, Facultad de Ciencias Sociales y Comunicaciones, Universidad Santo Tomas, Santiago 8370003, Chile; jtorresvallejos@santotomas.cl; 3 Escuela de Medicina, Universidad de Tarapacá, Arica 1000000, Chile; diegomarceloaragon@gmail.com

**Keywords:** migrant, immigrant, sleep disorders, insomnia, acculturation stress, acculturation, anxiety

## Abstract

Research on sleep disorders among migrant populations is limited, particularly in the context of south–south Latinos migrating to other Latin American countries. **Objective:** This study aims to analyze the effect that the acculturation process may play on the presence of the subjective assessment of insomnia symptoms in South American migrants in Chile. **Methods:** Under a cross-sectional design, 1844 South American migrants from Colombia, Venezuela and Peru, currently residing in Chile, were evaluated. Of these, 50% were women with an average age of 35 years. Data were collected using the Insomnia Severity Index (ISI), the EBEA scale for acculturation stress, a scale based on Berry’s acculturation strategies, and the anxiety subscale of the DASS-21. Mediation models were employed to assess anxiety as a mediating variable between both acculturation stress and acculturation orientations as well as insomnia symptoms. **Results:** A home-country-focused orientation was inversely related to insomnia symptoms, whereas a host-country-focused orientation showed a direct association. Anxiety did not mediate either of these relationships. However, acculturation stress was directly associated with the emergence of insomnia symptoms; in this relationship, anxiety partially mediated the negative effect of stress on sleep. **Conclusions:** There is a complex and dynamic interplay between the acculturation process, insomnia, and anxiety within south-to-south migration in Latin countries. Understanding these relationships could promote culturally sensitive interventions to mitigate the adverse effects of migration-related stressors on sleep health and the overall well-being of Latino migrants.

## 1. Introduction

Migration constitutes a critically relevant social phenomenon worldwide with over 281 million migrants across the globe reported in 2022 [1]. Substantial evidence indicates that moving to a new geographical area can affect migrants’ health and well-being particularly when prejudice and discrimination emerge in the host population [2,3,4]. These factors can not only have a detrimental effect on mental health but also in the emergence or worsening of other medical conditions [5]. In this context, most studies on the health impacts of Latino migration focus on south-to-north movement, in which individuals relocate from low-income countries to higher-income nations [6]. South–south migration is defined as the movement of individuals within low-income countries [7], and in the Latin American context, it is a relatively new and understudied phenomenon. For the case of Chile, although migration from neighboring countries has long existed, there was a substantial increase in the migrant population between 2017 and 2019 [8].

Our research group has previously studied the detrimental impact of discrimination on the general health and well-being of South American migrants [9,10,11]. South–south migration has substantial and fundamental differences from the south–north migration phenomenon. Unlike south–north migration, which often involves economic opportunities in higher-income countries, south–south migration frequently occurs between nations with comparable or limited economic resources. This results in restricted access to stable employment, formal housing, and social security [12]. Moreover, health and social support structures for migrants in south–south contexts tend to be less developed than those in high-income host countries, which can further exacerbate the impact of acculturation stress [13]. In addition, while cultural and linguistic similarities would facilitate acculturation in south–south migration, evidence suggests that intra-regional migrants may still face discrimination, social exclusion, or marginalization [14].

In this line of work, one potential yet understudied health consequence of the migratory process is the disruption of healthy sleep patterns, which are notably reflected in reduced sleep duration and/or sleep quality. Adequate sleep is an essential determinant of overall physical health, as insufficient sleep has been linked to an increased risk of hypertension, cardiovascular disorders, major cardiovascular events [15,16,17,18] and increased overall mortality [19]. The emergence of sleep disorders can be influenced by a wide spectrum of macro-social and individual factors, including socioeconomic status, gender, race/ethnicity, immigration status, and family or work-related conflicts [20]. Existing research on the relationship between migration and sleep disorders paints a complex and sometimes contradictory picture. While some studies suggest that immigrants may experience fewer sleep disturbances compared to native inhabitants of the host regions [21], these findings often apply to specific migrant subgroups, which are characterized by stronger linguistic acculturation [21], established residency, or relatively better health profiles [22]. In contrast, other studies have documented higher rates of sleep disorders among the immigrant groups, including African migrants in the United States [23], older adult migrants in China [24], refugee migrants and asylum seekers in Australia [25], immigrant Latinos in Spain [26], and refugees and non-Nordic migrants in Sweden [27,28]. In many of these contexts, poor sleep quality is linked to demographic, medical, and psychosocial variables. Among these factors, the most prominent are age, gender, chronic illness, anxiety, depression, migratory status, suboptimal living conditions, experiences of discrimination, challenges integrating into the host society, and lifestyle changes [29]. Notably, very few studies have addressed sleep disorders in South American migrants specifically. In this study, we have focused particularly on the perceived symptoms of insomnia given its higher prevalence.

Two frequently examined variables related to sleep disorders in the migrant population are acculturation strategies and acculturation stress. Acculturation strategies reflect a spectrum of attitudes, ranging from preserving one’s cultural heritage and identity to fully assimilating into the host culture [30]. These strategies also affect the choice to engage with or maintain distance from other social groups within the host society [31,32,33]. Prior research in South American contexts has linked acculturation strategies to various health physical and mental health indicators, such as blood cortisol levels [34] or life satisfaction [35]. However, their potential role in precipitating sleep disorders among South American migrants has not yet been explored.

Acculturation stress, another key factor associated with the acculturation process, emerges when the demands of adapting to a new cultural environment exceed an individual’s coping capacities, leading to adverse physical and emotional effects [36,37]. In South American migrants, this type of stress has been linked to lower psychological well-being [38], poorer mental health [39], and abnormal eating behaviors [40].

Given the limited empirical evidence on sleep disorders among migrant populations, especially in the context of south–south migration, this study aims to examine the influence of acculturation strategies and acculturation stress on sleep disorders, specifically on the perception of insomnia symptoms, in south–south Latino migrants. Furthermore, it explores whether anxiety mediates the relationship between these acculturation factors and the occurrence of insomnia symptoms.

## 2. Materials and Methods

### 2.1. Participants

Under a non-experimental, cross-sectional design, data were collected between August 2023 and February 2024. The sample comprises 1844 adult immigrants residing in Chile, who were distributed across the northern cities of Antofagasta (24.5%) and Arica (24.4%), the capital city, Santiago (26.7%) and the southern city of Temuco (24.4%). Among the participants, 33.1% identified as Colombian, 33% as Peruvian and 33.9% as Venezuelan. Regarding gender, 50.2% identified as female, and the average age was 35.02 years (SD = 10.84). As for migratory status, 63.5% of the samples indicated they were in a regular immigration situation, while 36.5% indicated that they were in a non-regular situation. Other sociodemographic characteristics of the participants can be found in Table 1.

### 2.2. Measures

#### 2.2.1. Insomnia [Outcome Variable]

Insomnia will be operationalized in accordance with the definition provided by the American Academy of Sleep Medicine [41], as the subjective perception of difficulty with sleep initiation, duration, consolidation, or quality, occurring despite adequate opportunity for sleep and resulting in some form of daytime impairment. The Spanish version of the Insomnia Severity Index (ISI) exploring 7 items and a score ranging from 0 to 28 points was utilized [42]. This version has demonstrated robust psychometric properties for the Spanish-speaking countries [43]. Scores obtained from the ISI categorize individuals in normal (0–7), subthreshold insomnia (8–14), clinical insomnia of moderate severity (15–21) and severe clinical insomnia (22–28). The reliability coefficients of this scale for this study were α = 0.921 and ω = 0.920.

#### 2.2.2. Acculturation Stress [Predictor Variable]

The brief scale for stress evaluation, EBEA, was employed in this study. This scale has also demonstrated favorable psychometric properties in terms of reliability and factorial structure for the Spanish-speaking countries [44]. The reliability coefficients of this scale for this study were α = 0.897 and ω = 0.889.

#### 2.2.3. Acculturation Orientation [Predictor Variable]

The Spanish adaptation of the Acculturation Strategies Scale developed by Zlobina et al. [45] was used. This instrument consists of 14 items with a Likert-type response format ranging from 1 to 5. The items are grouped into two dimensions: attitude toward the country of origin (α = 0.909 and ω = 0.909) and attitude toward the host country (α = 0.905 and ω = 0.904).

#### 2.2.4. Anxiety [Mediator Variable]

To assess anxiety, the ad hoc subfactor of the Depression, Anxiety and Stress Scale (DASS-21) [46,47,48] in its Spanish version [49] was utilized. The reliability coefficients of this subfactor for this study were α = 0.877 and ω = 0.876.

### 2.3. Procedures

This research is part of a larger project investigating salutogenic factors in a migrant population, which was approved by the scientific ethics committee of the Universidad Católica del Norte. The study was conducted in accordance with the ethical guidelines outlined in the Helsinki Declaration. Each participant identified through this methodology provided informed consent by signing a pre-participation consent form, which delineates the voluntary nature of their participation and guarantees their anonymity. As an inclusion criterion, participants were required to have resided in Chile for a period of more than three months, aligning with the duration of a tourist visa in the country. Participants were recruited using the snowball sampling technique. A basic sampling nucleus was established in each city, initiating with a seed sample in commercial establishments and places frequently attended by migrants in various cities in Chile (north, center and south of the country). The sample was progressively expanded as the original participants recommended or established connections with other like-minded individuals who met the characteristics sought.

### 2.4. Analytic Plan

A descriptive analysis of the demographic variables of the population was conducted. For the inferential analysis, reliability tests were performed. To explore data distribution, a Kolmogorov–Smirnov test with the Lilliefors correction was employed for each variable, revealing a non-normal data distribution in some instances. Correlations among sociodemographic variables such as age, gender, and legal situation were examined. Mediation analyses were conducted utilizing the Sobel Test, which establishes whether the indirect effect of the mediator significantly deviates from zero [50]. When the confidence interval excludes 0, the effect is deemed significant, and the mediating effect is confirmed. Note that although the same prejudice scale was applied in both groups, the target of prejudice differed. All direct and indirect relationships were estimated in the model, rendering it completely saturated, with zero degrees of freedom. According to Hayes [51], the statistical significance of indirect mediating effects is assessed through a bootstrap method, employing a point estimate of zero within a 95% bias-corrected and accelerated confidence interval (BCa CI). As such, a variable with a no-point estimate within zero interval is considered statistically significant. Thus, in the current study, BCa CI values were obtained from 5000 bootstrap samples. To comprehend the interplay among the variables of acculturation strategies, acculturation stress, depression, anxiety and stress, and finally insomnia, mediation analyses were conducted. Additionally, mediation analyses were controlled for gender (1 = male), age, and legal status in the country (1 = regular status). All data analyses were conducted using the statistical software Mplus 8.10 [52].

## 3. Results

Descriptive statistics and correlations between variables are shown in Table 2.

**Figure 1:**
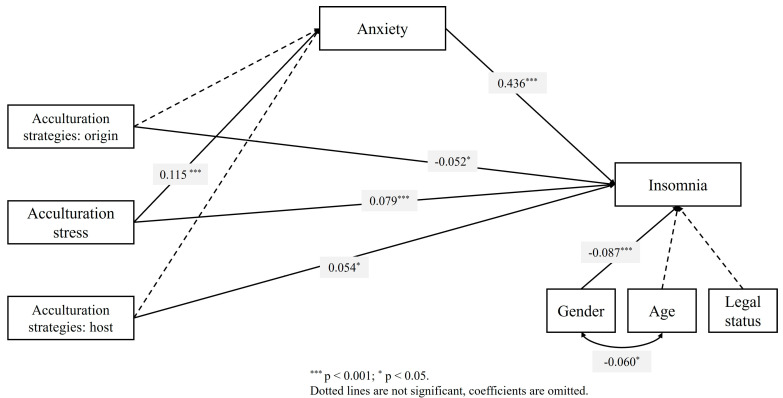
Mediation model for acculturation stress, strategies, and insomnia for the whole sample.

The results showed a partial mediation effect through acculturation stress, wherein the total effect (β = 0.129, *p* < 0.001) was attenuated when the mediator variable anxiety was introduced (direct effect: β = 0.078, *p* < 0.05). Moreover, the indirect effect was statistically significant. Conversely, acculturation strategies pertaining to both origin and host cultures did not exhibit significant indirect effects through the relationship between independent and dependent variables. The purposed model (see Figure 1) explained 21.7% of the total variance. Notably, only gender displayed a significant effect as an insomnia control variable.

The total, direct and indirect effects are shown in Table 3.

## 4. Discussion

Acculturation and sleep disorders constitute a multidimensional phenomenon influenced by cultural, psychological, and social factors across diverse migrant contexts. South–south migration stands out as a distinctive phenomenon within Latin America due to the unique geopolitical and economic contexts shaping mobility in the region. While shared language and cultural practices can facilitate certain aspects of acculturation, persistent inequalities, political instability, and varying economic opportunities also influence migration flows. In this sense, migrants often traverse complex pathways involving both intra-regional cooperation and localized tensions, which markedly differ from the classic south–north migration model. Considering the dynamic and evolving landscape of south–south migration among Latino populations (especially between 2017 to 2019, when massive south-south Latino migration occurred), examining the role of acculturation in shaping sleep patterns emerges as a crucial area of inquiry. Our findings suggest that acculturation orientations exert an influence on the subjective perception of sleep difficulties, including issues related to sleep initiation, duration, consolidation, and quality. Specifically, individuals who maintained stronger ties to their culture of origin reported fewer sleep difficulties, whereas those who more fully adopted the host culture experienced more insomnia symptoms. Notably, these relationships were not explained by anxiety levels.

Although research on how acculturation strategies might affect sleep patterns is relatively limited, these findings are in alignment with similar studies in the North American and European context. While south–south migration differs substantially from south–north migration, evidence shows that migrants who more thoroughly integrate into the host culture often report poorer sleep outcomes. For instance, Portuguese and Moroccan migrant women in Germany who were reportedly more integrated showed poorer sleep quality than those who were less integrated [53]. Similarly, in the United States, migrant women with higher levels of linguistic acculturation were more prone to sleep disturbances with linguistic acculturation mediating the link between immigrant status and sleep complaints [21]. In alignment, Mexican migrants in the United States who predominantly spoke English reported shorter sleep durations than those who primarily spoke Spanish [54]. One possible driver of this phenomenon is that adopting a new cultural identity often demands heightened vigilance, requiring individuals to continuously seek information about norms, language, or customs of the host culture [55]. This constant monitoring might lead to a state of cognitive-–emotional hyperarousal that in turn has been linked to a greater risk of developing primary insomnia [56]. Similar patterns may help explain the reported impact on sleep quality among south–south Latino migrants who more fully integrate into their host culture. Despite certain commonalities in language, these migrants often confront distinct local customs, social hierarchies, or community expectations that can heighten pressure to assimilate. At the same time, moving away from familiar support networks and routines could introduce distinct sources of chronic stress that ultimately may interfere with sleep quality. The latter is further supported by studies finding that anxiety, a known risk factor for sleep disorders, did not mediate acculturation orientation, suggesting it may not be central to the individual’s coping mechanism. This would reinforce the hypothesis that individuals who adjust more thoroughly to the host culture may experience a state of hypervigilance related to their acculturation orientation, which would differ from anxiety symptoms, thereby differentiating their sleep disturbance from anxiety-driven insomnia. This aligns with previous research that suggests that the preservation of cultural heritage can act as a protective factor against mental health problems, including anxious and depressive symptoms, and is linked to more favorable indicators of well-being [35]. In addition, another possible hypothesis is that maintaining the customs and cultures of the place of origin enhances the identity (ethnic or national) of the participants, which is a factor that has been reported to be protective of mental health and well-being in migrant populations and thus could have some effect on this relationship [41]

As for acculturation stress, our findings suggest that acculturation stress exerts a direct effect on insomnia symptoms where higher levels of acculturation stress correlate with more severe insomnia complaints. This relationship was partially mediated by anxiety, mirroring similar findings among Latino migrants in the United States [57]. Given that both acculturation stress and sleep difficulties can amplify depressive symptoms among certain subgroups [58], the broader health implications of disrupted sleep are substantial. While some studies have yielded mixed results regarding the link between acculturation stress and sleep [59], larger investigations have increasingly highlighted acculturation stress as a core driver of sleep health disparities among migrants [60]. Indeed, our findings suggest that this association holds whether migrants relocate to a culturally similar or dissimilar host society.

Moreover, socioeconomic status, educational level, and employment status can also influence sleep disturbances among Latino migrant populations [61]. For instance, limited financial resources can constrain access to stable housing, leading to overcrowded or otherwise unsuitable living conditions that can directly affect sleep quality [62]. Additionally, lower educational levels may impede the ability to access better employment opportunities, access mental health support, or advocate for better living conditions [63]. Meanwhile, employment status or precarious employment conditions with irregular sources of income can also further fuel acculturation stress and sleep disturbances in the Latino migrant population [64].

Although acculturation strategies, acculturation stress, anxiety, insomnia, socioeconomic status, educational level, and employment status intersect with the broader challenges of the migration experience, the latter three variables were not examined in the present study, constituting a notable limitation. Further limitations arise from the snowball sampling approach employed in data collection. This method can introduce selection bias, as individuals within the same social networks often share similar characteristics and experiences, thereby reducing the generalizability of the results to broader migrant populations in south–south migration contexts. In addition, the cross-sectional design restricts the findings to a single point in time, preventing any causal inferences about how acculturation factors, sleep disturbances, and anxiety may interact throughout the acculturation process. Nonetheless, the large sample size employed reinforces the reliability and representativeness of the findings, and the use of well-validated, culturally sensitive scales to assess acculturation strategies, acculturation stress, and sleep disturbances strengthens the measurement process. An important limitation is that only the subjective assessment of sleep disorders was considered—in this case, of symptoms associated with insomnia—so future studies could incorporate measurements such as polysomnography or actinography. Although this study did not consider the presence of severe psychiatric disorders as an exclusion criterion, people with such conditions did not participate. However, we did not control the presence of physical illnesses that might be associated with an increased presence of insomnia symptoms, such as chronic pain or cancer, which could also be controlled for future research.

Our findings should therefore be interpreted with caution, as they only provide an initial exploration of potential mediating relationships rather than definitive causal evidence. Employing theoretical coherence and empirical covariation can strengthen causal inferences drawn from cross-sectional data [65].

Finally, the findings of this study highlight the importance of addressing both psychological and cultural factors in promoting healthier sleep patterns among south–south Latino migrants. By recognizing that acculturation stress and cultural orientations can significantly impact sleep quality, policymakers can design more effective and culturally informed programs. This could include implementing community-based interventions that foster cultural preservation while promoting host society integration as well as offering targeted mental health services, including sleep assessments and behavioral strategies for insomnia. Ultimately, a holistic focus on sleep quality, mental well-being, and cultural identity can strengthen integration and promote overall health among south–south Latino migrants. Although south–south migration in Latin America has long existed, it has recently experienced a marked surge, making it a highly dynamic phenomenon in need of further empirical exploration. Future research would benefit from adopting longitudinal designs, capturing the evolving interplay between acculturation factors, sleep disturbances, and associated mental health outcomes over time. Moreover, investigations focusing on specific subpopulations, such as older adults, pregnant women, or migrants with pre-existing health conditions, might also provide a deeper understanding on how distinct demographic, psychosocial, or cultural factors influence sleep health in relation to the south-south migratory patterns in the Latin American context.

## 5. Conclusions

This study underscores the intricate interplay among acculturation stress, insomnia, and anxiety within the context of south-to-south migration among Latino migrants in South American countries. The findings reveal a significant association between the acculturation process and sleep disorders. Particularly, we found that preserving the home country’s cultural heritage exerts an inverse effect on the presence of sleep disorders, whereas a host country orientation is directly associated with insomnia symptoms. Interestingly, anxiety did not mediate these relationships. Moreover, regarding acculturation stress, higher levels of stress are linked to increased insomnia symptoms with anxiety playing a pivotal role in this relationship. These insights underscore the complex dynamics underlying the acculturation process and its implications for sleep health among migrant populations. Understanding these implications may be valuable to effectively address the adverse effects of migration-related stressors on sleep health and overall well-being and promoting culturally sensitive interventions to mitigate said effects. Such initiatives could play a crucial role in safeguarding the health and resilience of migrant communities in the South American migration process, ultimately fostering social integration and equitable health outcomes.

## Figures and Tables

**Table 1:** Sociodemographic characteristics of the participants.

Variable	Category	Total (*n* = 1844), *n* (%)
Years in Chile	<5 years	1132 (61.4)
	6–10 years	463 (25.1)
	>10 years	247 (13.4)
	No answer	2 (0.1)
Occupation	Employee	1409 (76.4)
	Retired	22 (1.2)
	Unemployed	220 (11.9)
	Household worker	99 (5.4)
	Student	67 (3.6)
	No answer	27 (1.5)
Educational level	Incomplete primary education	61 (3.3)
	Primary education	233 (12.6)
	Secondary education	645 (35)
	Incomplete technical education	199 (10.8)
	Technical education	305 (16.5)
	Incomplete university education	131 (7.1)
	University education	230 (12.5)
	Postgraduate	36 (2)
	No answer	4 (0.2)
Income	<USD 125	140 (7.6)
	USD 126–375	363 (19.7)
	USD 376–750	824 (44.7)
	USD 751–1250	334 (18.1)
	USD 1251–1875	102 (5.5)
	>USD 1876	67 (3.6)
	No answer	14 (0.8)

**Table 2:** Means (M), standard deviations (SD), and correlations across all variables for the total sample.

	M	SD	1	2	3	4	5	6	7
1. Age (years)	35.03	10.84	–						
2. Gender (1 = male)	49.8%		−0.058 *	–					
3. Legal situation (1 = regular)	63.5%		0.142 ***	−0.091 ***	–				
4. Insomnia	7.59	6.00	−0.060 **	−0.076 **	−0.050 *	–			
5. Anxiety	0.65	0.64	−0.133 ***	0.015	−0.184 ***	0.441 ***	–		
6. Acculturation strategies: culture of origin	3.79	0.89	0.024	−0.050 *	−0.059 *	−0.051 *	−0.019	–	
7. Acculturation strategies: host culture	2.99	0.85	0.026	−0.051 *	0.079 **	0.023	−0.051 *	0.101 ***	–
8. Acculturation stress	3.68	1.07	−0.094 ***	0.081 ***	−0.028	0.116 ***	0.119 ***	0.014	−0.118 ***

Note. *** *p* < 0.001. ** *p* < 0.01. * *p* < 0.05.

**Table 3:** Completely standardized indirect effects for the mediation model.

Variables	Direct Effect β [95% C.I.]	Indirect Effect β [95% C.I.]	Total Effect β [95% C.I.]
Acculturation strategies: origin culture	−0.052 * [−0.097, −0.013]	−0.007 [−0.027, −0.013]	−0.060 * [−0.110, −0.015]
Acculturation strategies: host culture	0.054 * [0.011, 0.098]	−0.016 [−0.038, 0.005]	0.038 [−0.010, 0.086]
Acculturation stress	0.079 *** [0.037, 0.119]	0.050 *** [0.031, 0.070]	0.129 *** [0.082, 0.172]

Note. *** *p* < 0.001. * *p* < 0.05.

## Data Availability

Data and materials will be made available upon reasonable request to the corresponding author.
